# Supplementary material for: Validation of L-type calcium channel blocker amlodipine as a novel ADHD treatment through cross-species analysis, drug-target Mendelian randomization, and clinical evidence from medical records
Source: Neuropsychopharmacology. 2025 Feb 14;50(7):1145–55. doi: 10.1038/s41386-025-02062-x (PMC12089589; doi:10.1038/s41386-025-02062-x)
Supplement: Supplementary file 1 — Supplementary information [file 41386_2025_2062_MOESM1_ESM.docx]

**Supplementary Information**

**Supplementary figures**


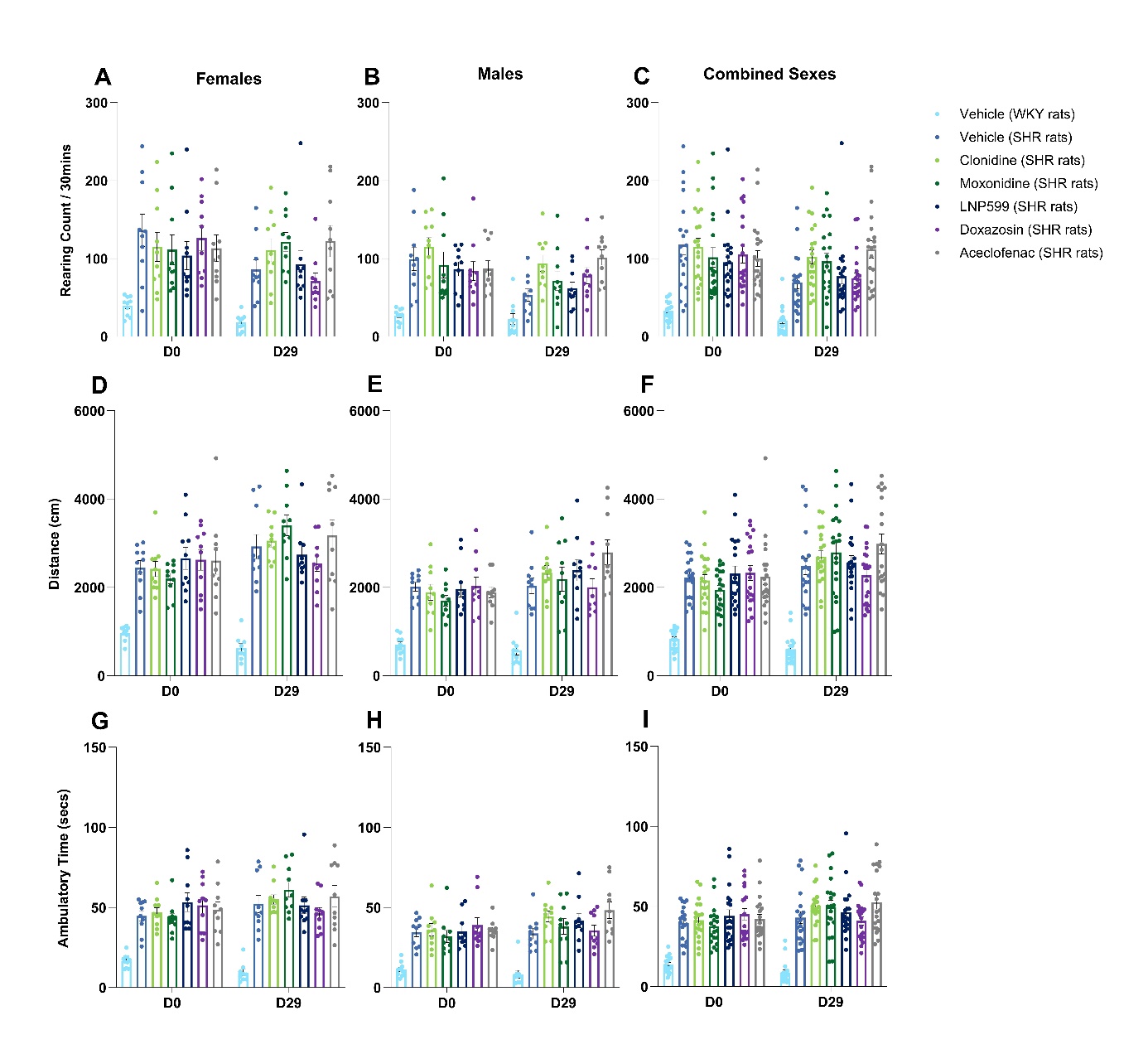
**Supplementary figure 1. Drug effect on Open Field Behavior in SHR rats.** SHR rats were treated with either clonidine, moxonidine, LNP599, doxazosin, aceclofenac or a vehicle daily for 30 days; WKY rats received vehicle only. Assessments were made before treatment (D0) and at end of treatment (D29) using the OFT. Measurements included rearing frequency (A, B, C), distance traveled in the center zone (D, E, F), and ambulatory time in the center zone (G, H, I). No significant reduction of rearing frequency, distance traveled, or ambulatory time was observed at either time points, day 0 or day 29, between vehicle-treated SHR rats and those treated with five different drugs. This finding was consistent across female, male, and combined sexes. Data are mean ± SEM, n = 10, a mixed-model approach (REML), followed by a post-hoc analysis with Fisher’s LSD test without correction and Tukey’s multiple comparisons tests.


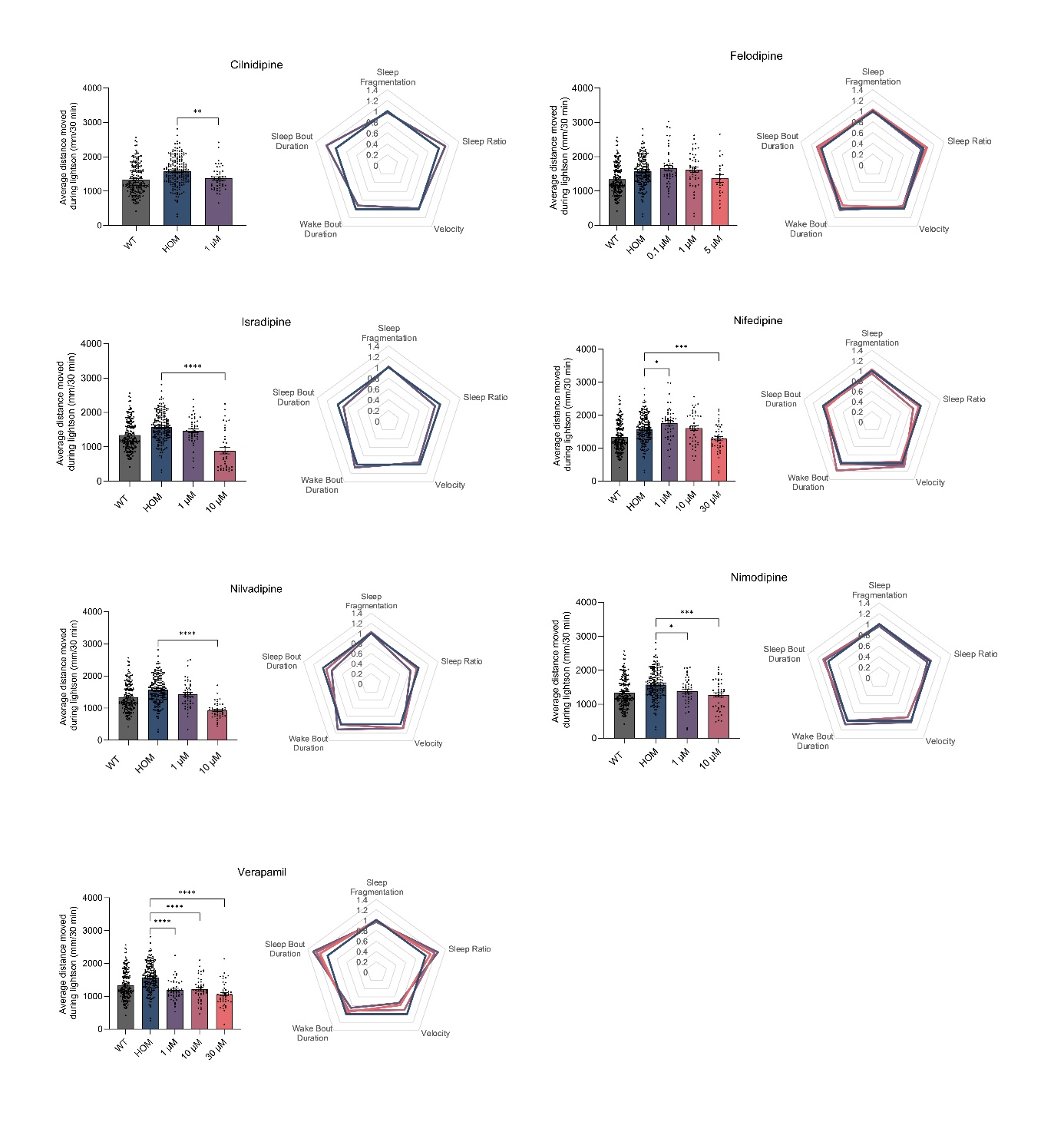


**Supplementary figure 2. Effects of L-Type Calcium Channel Blockers on hyperactivity and sleep parameters in larval *adgrl3.1^-/-^* zebrafish.** *Adgrl3.1^-/-^* larvae were treated with 12 LTCCBs: cilnidipine, felodipine, isradipine, nifedipine, nilvadipine, nimodipine and verapamil, shown here, and diltiazem, lacidipine, nicardipine, nisoldipine and nitrendipine (n.s. not shown).  All drugs were given at three different concentrations (1 µM, 10 µM, and 30 µM, felodipine at: 0.1 µM, 1 µM and 5 µM) and compared to larvae treated with 0.3% DMSO. Average distance moved during five 30-min periods of lights-on and sleep parameters during the night were measured and analyzed (for detailed methods see Sveinsdóttir *et al.*[^1^](https://paperpile.com/c/pGLcgE/bmod)). Significant reduction in distance moved was observed for cilnidipine (1 µM), isradipine (10 µM), nifedipine (30 µM), nilvadipine (10 µM), nimodipine (1 µM and 10 µM) and verapamil (1 µM, 10 µM and 30 µM). Five sleep parameters were analyzed, sleep fragmentation, sleep ratio, velocity, wake bout duration and sleep bout duration. LTCCBs that did reduce hyperactivity had minor effects on sleep parameters, apart from verapamil which induced higher sleep ratio and higher average sleep bout duration. Cilnidipine, felodipine, isradipine, nilvadipine and nimodipine induce toxicity at medium to high doses. WT larvae treated with 0.3% DMSO are included for visual comparison. Data are mean ± SEM., n = 48, one-way ANOVA and Dunnett’s multiple comparison post hoc analysis, **P* ≤ 0.05, ***P* ≤ 0.01, ****P* ≤ 0.001, *****P* ≤ 0.0001.


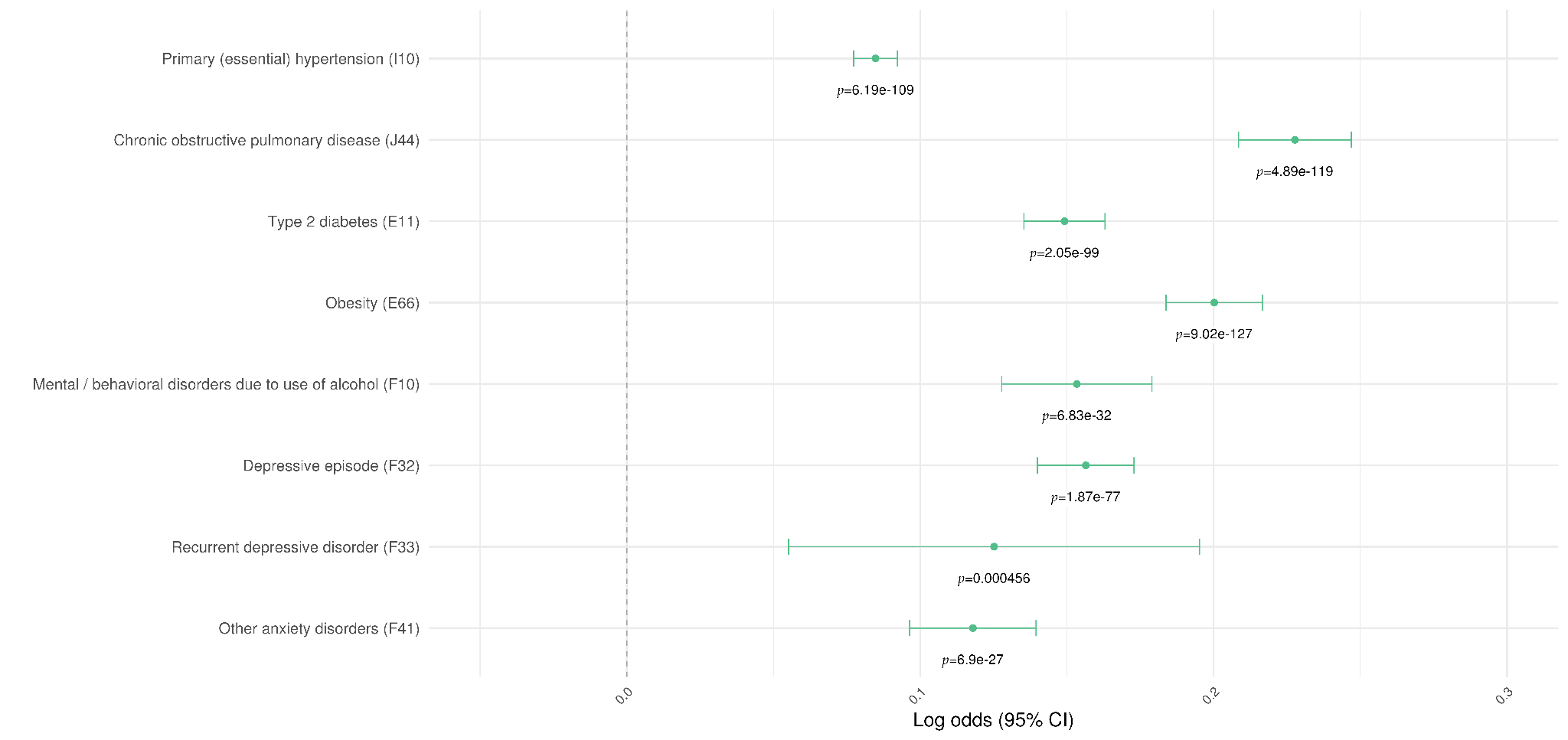


**Supplementary Figure 3. UK Biobank inpatient diagnosis as a function of ADHD risk score.** Each row represents a logistic regression model where the UK Biobank inpatient diagnoses in the left-hand column (with ICD-10 codes in parentheses) are modeled as functions of ADHD risk score. Points provide the coefficient estimates for this predictor with the corresponding p-values printed below. The bars around these points represent 95% confidence intervals for these estimates. Points and confidence interval bars in green indicate that the given predictor is significant (*p* < 0.05) in the given model.


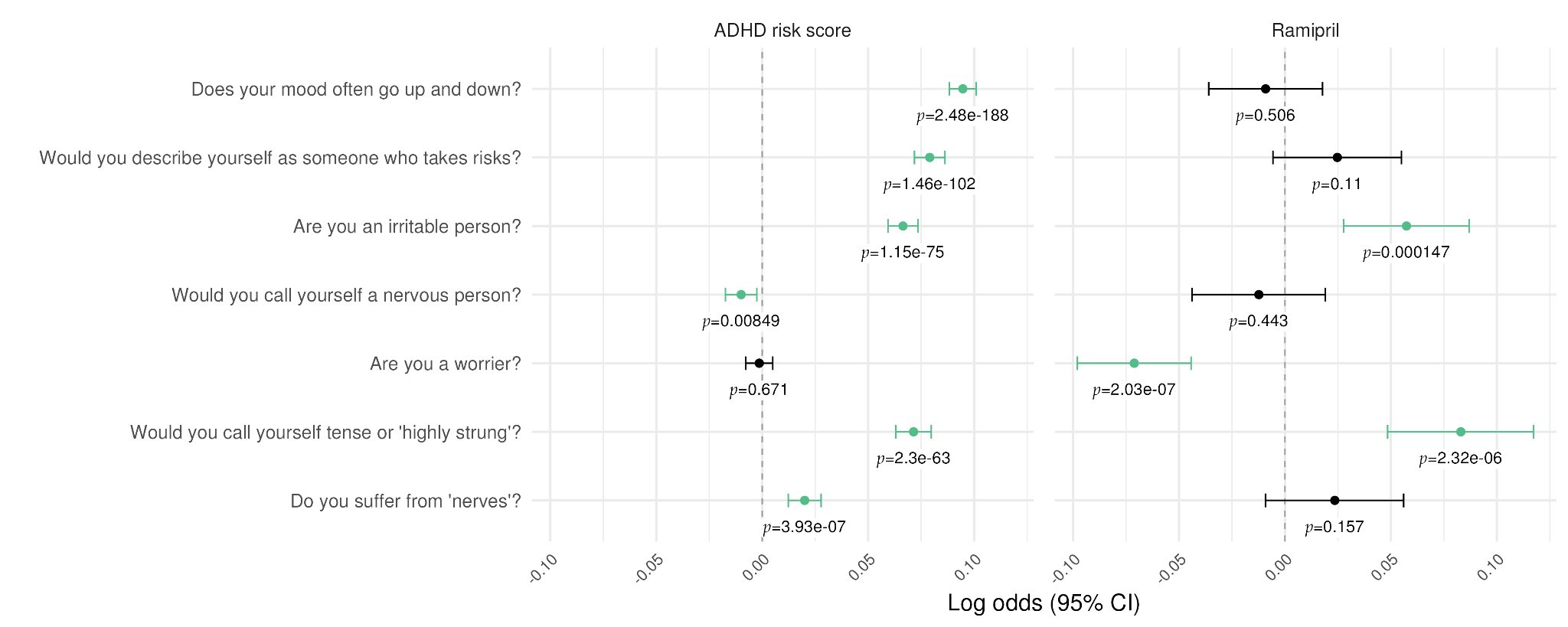


**Supplementary Figure 4. UK Biobank mental health questionnaire responses as functions of ADHD risk score and ramipril prescription status.** Each row represents a logistic regression model where UK Biobank participants’ responses to the question in the left-hand column are modeled as functions of ADHD risk score and amlodipine prescription status. The points plotted in the center and right-hand columns are the coefficient estimates for these two predictors with their corresponding p-values printed below. The bars around these points represent 95% confidence intervals for these estimates. Points and confidence interval bars in green indicate that the given predictor is significant (*p* < 0.05) in the given model.

**Supplementary References**

1. [Sveinsdóttir, H. S. *et al.* Novel non-stimulants rescue hyperactive phenotype in an adgrl3.1 mutant zebrafish model of ADHD. *Neuropsychopharmacology* **48**, 1155–1163 (2023).](http://paperpile.com/b/pGLcgE/bmod)
